# GESPA: classifying nsSNPs to predict disease association

**DOI:** 10.1186/s12859-015-0673-2

**Published:** 2015-07-25

**Authors:** Jay K. Khurana, Jay E. Reeder, Antony E. Shrimpton, Juilee Thakar

**Affiliations:** 1 Department of Urology, SUNY Upstate Medical University, Syracuse, NY USA; 2 Department of Obstetrics and Gynecology, University of Rochester, Rochester, NY USA; 3 Department of Pathology, SUNY Upstate Medical University, Syracuse, NY USA; 4 Department of Microbiology and Immunology, University of Rochester, Rochester, NY USA; 5 Department of Biostatistics and Computational Biology, University of Rochester, Rochester, NY USA

**Keywords:** Genetic disease, Single nucleotide polymorphisms, nsSNP prediction, SNP phenotype, SNP pathogenicity, Next Generation Sequencing

## Abstract

**Background:**

Non-synonymous single nucleotide polymorphisms (nsSNPs) are the most common DNA sequence variation associated with disease in humans. Thus determining the clinical significance of each nsSNP is of great importance. Potential detrimental nsSNPs may be identified by genetic association studies or by functional analysis in the laboratory, both of which are expensive and time consuming. Existing computational methods lack accuracy and features to facilitate nsSNP classification for clinical use. We developed the GESPA (GEnomic Single nucleotide Polymorphism Analyzer) program to predict the pathogenicity and disease phenotype of nsSNPs.

**Results:**

GESPA is a user-friendly software package for classifying disease association of nsSNPs. It allows flexibility in acceptable input formats and predicts the pathogenicity of a given nsSNP by assessing the conservation of amino acids in orthologs and paralogs and supplementing this information with data from medical literature. The development and testing of GESPA was performed using the humsavar, ClinVar and humvar datasets. Additionally, GESPA also predicts the disease phenotype associated with a nsSNP with high accuracy, a feature unavailable in existing software. GESPA’s overall accuracy exceeds existing computational methods for predicting nsSNP pathogenicity. The usability of GESPA is enhanced by fast SQL-based cloud storage and retrieval of data.

**Conclusions:**

GESPA is a novel bioinformatics tool to determine the pathogenicity and phenotypes of nsSNPs. We anticipate that GESPA will become a useful clinical framework for predicting the disease association of nsSNPs. The program, executable jar file, source code, GPL 3.0 license, user guide, and test data with instructions are available at http://sourceforge.net/projects/gespa.

**Electronic supplementary material:**

The online version of this article (doi:10.1186/s12859-015-0673-2) contains supplementary material, which is available to authorized users.

## Background

Non-synonymous single nucleotide polymorphisms (nsSNPs) frequently show allele specific functional differences and linkage to genetic disease because of the change in primary protein sequence [1]. Unraveling their clinical significance will lead to major strides within the field of medical genetics. The potential of next generation sequencing (NGS) in disease diagnosis demands a process that will allow for more nsSNPs to be identified as potential risk factors in genetic conditions. NGS sequencing for personalized medicine is rapidly becoming routine [2, 3] and is expected to replace other methods for SNP analysis which are more labor-intensive, such as PCR followed by allele specific oligonucleotide hybridization [4], reverse dot-blot hybridization [5] and fragment length polymorphism analysis [6], which are slower and difficult to scale up for whole genome analysis [7].

Determination of the pathogenicity of nsSNPs by functional analysis or genetic association studies is time consuming and costly. Several computational strategies have been developed to identify deleterious nsSNPs. PolyPhen [1] and PolyPhen 2 [8] calculate a Position Specific Independent Count (PSIC) score [9] and combine this value with structural and biochemical considerations to predict pathogenicity. SIFT [10] uses homology comparisons through sequence alignments to reach a prediction of pathogenicity. Additional methods relying on similar evolutionary variables and statistical techniques to predict nsSNP pathogenicity have been described [11–20]. However, they yield comparable or lower accuracies than the aforementioned tools without predicting a distinct phenotype. Several reviews of the current technology and methods used to detect disease-linked nsSNPs such as PolyPhen 2 and SIFT have found low accuracy rates [21–24]. Furthermore, existing programs all lack a flexibility of acceptable input formats, often requiring information not immediately available to researchers such as protein sequences [25] or multiple sequence alignments [26] while not readily allowing input formats as common as nucleotide locations or dbSNP [27] accession numbers. These programs are also limited significantly by the scope of the information they access and make available to users.

We have developed a novel bioinformatics tool, GESPA (GEnomic Single nucleotide Polymorphism Analyzer) which addresses the drawbacks of previous software. Particularly, the software predicts the pathogenicity of a given nsSNP by assessing the conservation of amino acids in homologs and paralogs and supplementing this information with data from medical literature. In addition, GESPA is the first program to predict nsSNP phenotype and does so by assessing other nsSNPs in the potential genetic functional hotspot in which a given nsSNP resides. GESPA’s interface is intuitive, accepts nsSNPs to be entered in several different formats and rapidly displays pathogenicity. Moreover, GESPA produces detailed reports for further studies. Thus, GESPA can be used to analyze NGS data and design future studies.

## Implementation

### Input format

We have provided great versatility in user input by accepting nsSNPs’s protein location in the NCBI RefSeq, dbSNP accession number, nucleotide location in the RefSeq, and flanking nucleotide sequence in the RefSeq (starting with the nucleotide at which the mutation occurs). Examples of these input types are available in the user manual available at GESPA’s sourceforge website (see Availability and Requirements). We have also provided the additional option of uploading a batch file containing multiple genes and related nsSNPs. Sample batch files and guidelines for formatting them are also available in the user manual. GESPA does not focus on non-coding variants because of lower resolution and association with subclinical diseases [24].

### Gene specific information retrieval

GESPA uses multiple sequence alignments of paralogous and orthologous genes with the assumption that the mutations in the conserved region will be more detrimental than in the non-conserved region [1, 8]. Moreover, GESPA also uses medical literature in its assessment of nsSNPs. The HUGO gene symbol of a protein coding gene is first used to obtain the relevant information from NCBI databases, namely Entrez Gene [28], ClinVar [29], GenBank [30], and HomoloGene. Entrez Gene is then used for collection of relevant information such as accession numbers and annotations. Next, GenBank is used to retrieve sequences by accession numbers. The HomoloGene database is used to obtain genome-wide orthologous sequences based on pre-computed BLASTP alignments [31] that have been found to be critical in predicting nsSNP pathogenicity [32]. Data was retrieved using the NCBI Eutils and HtmlUnit, a JavaScript enabled browser without a graphical user interface.

**Fig. 1:**
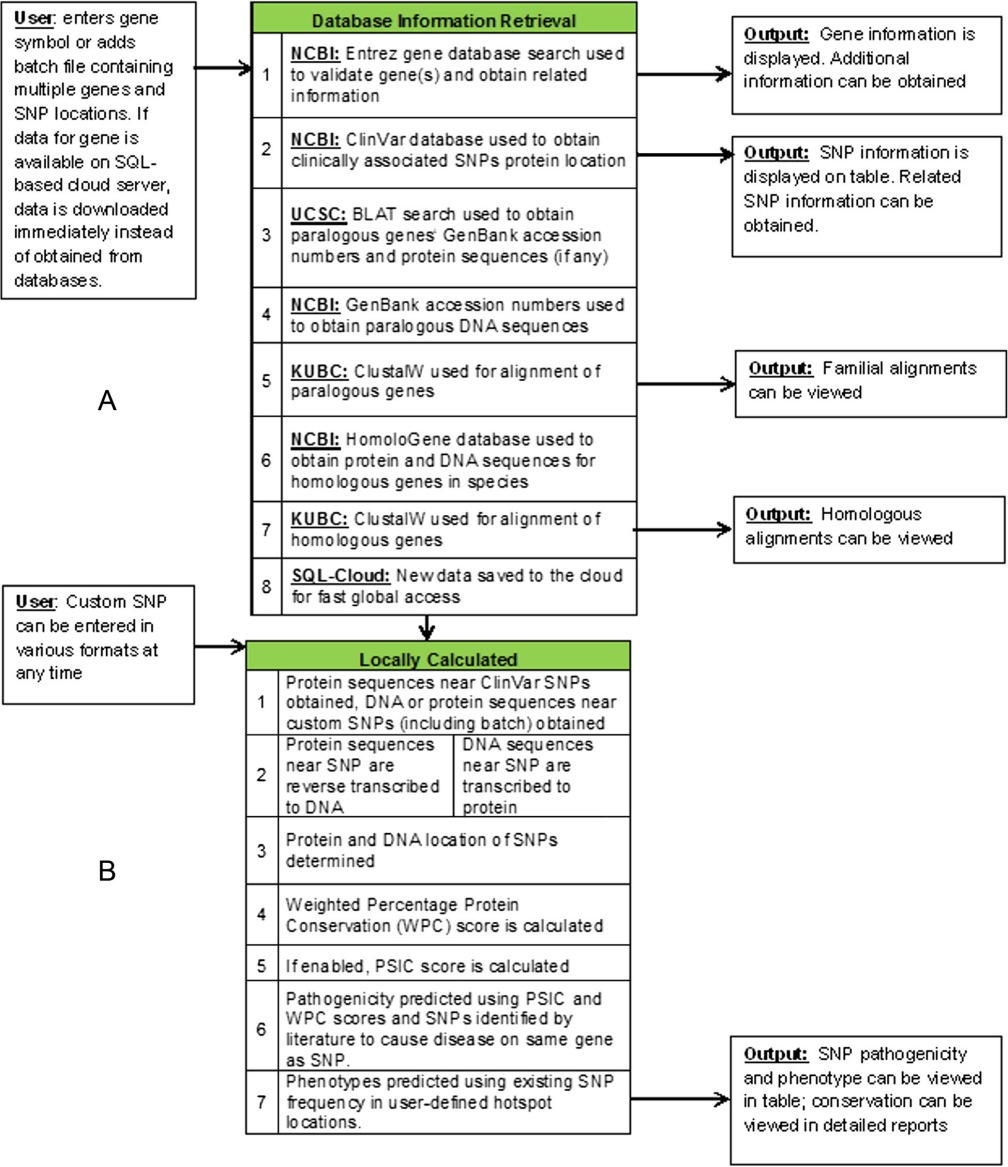
Flowchart overview of algorithm used in GESPA. **a** Input, output and retrieval of the data from available resources. Left cells show acceptable inputs including a HUGO symbol or a batch file which contains multiple genes and nsSNPs of interest. The middle cells represent resources used to obtain required information. Genes with data already saved on custom SQL-based server cloud are downloaded instantly, circumventing slower retrieval from databases. The right cells represent output obtained from each resource. **b** Steps involved in nsSNP pathogenicity and phenotype prediction algorithm. The steps described in the methods section are listed in the middle table which will determine the pathogenicity and phenotype of the given nsSNP. The results are available in the table format

The incorporation of paralogs improved the ability to detect detrimental mutations since they maintain a high level of conservation within species [32]. The GenBank accession numbers for all paralogs (including isoforms and splice variants) with a low BLAT e-value score, i.e., highly conserved paralogs, are obtained from the UCSC BLAT search tool [33]. The paralogous sequences themselves are subsequently obtained using GenBank. Paralogs and orthologs are compiled into two separate multiple sequence alignments for the user using the Kyoto University Bioinformatics Center’s (KUBC) ClustalW tool [34]. Note that paralogs and orthologs are used together in one alignment for pathogenicity prediction algorithm. The retrieval of gene-specific information is summarized in Fig. 1a.

### Evaluation of conservation underlying DNA and protein sequences

GESPA uses DNA and protein sequences to estimate conservation and either one of them can be used as inputs. In order to find conservation of the DNA sequence underlying a given protein, the amino acid sequence is translated in four steps. (1) A sequence of ten amino acids starting with the amino acid at the location of the nsSNP is obtained using the RefSeq protein sequence. In the event that the amino acid of the nsSNP is one of the last ten amino acids, the algorithm is performed backwards. (2) For each amino acid, all of the combinations of nucleotides (codons) are searched in the nucleotide RefSeq until a matching position is found. The speed of the process is increased by using known codon frequencies in the human genome. (3) The first three nucleotides from the matching position are compared. If an amino acid has been changed then the location of the mutated nucleotide is provided along with the nsSNP DNA codon, and conservation in DNA alignments. (4) Finally, conservation is calculated by using the location of the nucleotide that caused the nsSNP using corresponding DNA orthologous and paralogous multiple sequence alignments. The percent conservation is also provided to the user. In a few rare cases, more than one possible nucleotide change in the original amino acid codon could result in the changed amino acid, in which case a definitive DNA location and conservation cannot be determined.

If the nsSNP is entered with a DNA location, the underlying protein conservation is determined. Particularly, a sequence of 100 nucleotides (for unique identification) from the start of the location specified or a user-provided DNA flanking sequence is converted into amino acids using the appropriate reading frame. The protein RefSeq id of the correctly translated amino acid sequence is used to obtain coordinates along with the identity of the mutated amino acid (determined if user enters mutated nucleotide). Next, PSIC score [9] and Weighted Protein Conservation (WPC) score (as described below) are calculated.

To calculate the Weighted Protein Conservation (WPC) score, the location of the nsSNP of interest is used to find the corresponding positions in orthologous and paralogous amino acid alignments. At the corresponding positions, the number of sequences with amino acids matching the human variant in the alignments is determined. The WPC score given below is subsequently calculated by dividing this number by the total number of valid sequences.

1 $$ WP{C}_i={a}_i/{n}_h $$

Where, i is the position of the amino acid, *a*_*i*_ is the number of amino acids at position i in homologous (h) alignments (both paralogous and orthologous) that match the unmutated human amino acid at position i and n_h_ is the number of homologous sequences (both paralogous and orthologous) in the given alignment. It should be noted that the WPC score does not measure the overall conservation of a location in the alignments but rather the conservation of the corresponding nsSNP amino acid in the unchanged human gene of interest.

### Determination of phenotype and pathogenicity

In order to determine phenotype, GESPA evaluates frequencies of disease-associated nsSNPs in a user specified physical distance from the nsSNP using the ClinVar database. The regions with high frequencies of disease-associated nsSNPs are known as functional hotspots which have been linked to similar phenotypes and SNP pathogenicity [35, 36]. GESPA uses potential functional hotspots to determine the frequency of phenotypes in a user specified range. The phenotype with the highest frequency is predicted to be the phenotype of the nsSNP.

The pathogenicity of mutations is determined by evaluating functional hotspots and then calculating the PSIC score [9] and/or WPC score. Specifically, if the nsSNP of interest is determined to *not* be located in a functional hotspot then it is predicted to be benign. Note that functional hotspots are broadly defined so that existence of any number of known disease-associated nsSNPs is considered as a functional hotspot. This leads to higher confidence in predicting benign nsSNPs not located in potential functional hotspots. The functional hotspot feature can be turned off if the user is interested in *de novo* variants on genes previously largely ignored by the literature or variants which are not observed in the reference populations. Phenotype cannot be predicted for these variants. In such cases, evaluation is based on PSIC score [9] and/or WPC score.

All nsSNPs with stop-gained mutations are predicted to be pathogenic as long as they are further than 50 nucleotides from the start of the final intron. Remaining nsSNPs in *potential* functional hotspots which have a PSIC Score below 1.03 or a WPC score below 40 are classified as benign while SNPs with a WPC score ≥ to 40 are classified pathogenic.

These thresholds were determined by training and testing the pathogenicity prediction algorithm using humsavar (test set), ClinVar (data source) and humvar (training set) datasets. The entire process of local calculations performed by GESPA is summarized in Fig. 1b. The assessment of pathogenicity prediction was performed by using a cross-validation method on the humsavar dataset and through using the humvar dataset as a training set and the humsavar as a test set. The feature of assessing stop-gained mutations was disabled during assessment.

GESPA’s performance was compared with some of the most popular nsSNP pathogenicity classification tools (Table 1). These tools had their algorithm cutpoints tested and optimized in Choi *et al.* [37]. These optimal cutpoints were used by Choi *et al.* to test the sensitivity, specificity, and balanced accuracy of the programs on the humsavar dataset. GESPA’s performance was assessed by using the same procedure and data-sets published in Choi *et al.*

**Table 1:** GESPA pathogenicity classifier accuracy compared to other software using humsavar test set

Software	Sensitivity (%)^a^	Specificity (%)^b^	Balanced accuracy (%)^c^	ROC Curve AUC
GESPA (humsavar cross validation)	96.41	79.49	87.95	0.936
GESPA (humvar training set)	96.31	79.23	87.78	0.932
Polyphen 2	88.68	62.45	75.56	0.847
SIFT	85.03	68.95	76.99	0.854
PROVEAN	78.39	79.11	78.75	0.848
Mutation Assessor	85.29	71.02	78.15	0.848

^a^Sensitivity = TP/(TP + FN)

^b^Specificity = TN/(TN + FP)

^c^Balanced Accuracy = (Sensitivity + Specificity)/2

### SQL-based cloud server integration

In order to facilitate rapid global retrieval of all data (sequences, alignments, and nsSNPs from medical literature) as well as to ensure that the data is consistently updated, we use an SQL-based cloud server as a framework for pre-computed data retrieval. Once all data is collected for a particular gene, it is saved to the cloud allowing for faster user accession. Moreover, the data is time stamped and users have the option of selecting the data based on its time stamp. If data on the cloud is found to be older than the user specified threshold, data is retrieved from the most current version of the databases and replaces the older version on the cloud.

### User interface

**Fig. 2:**
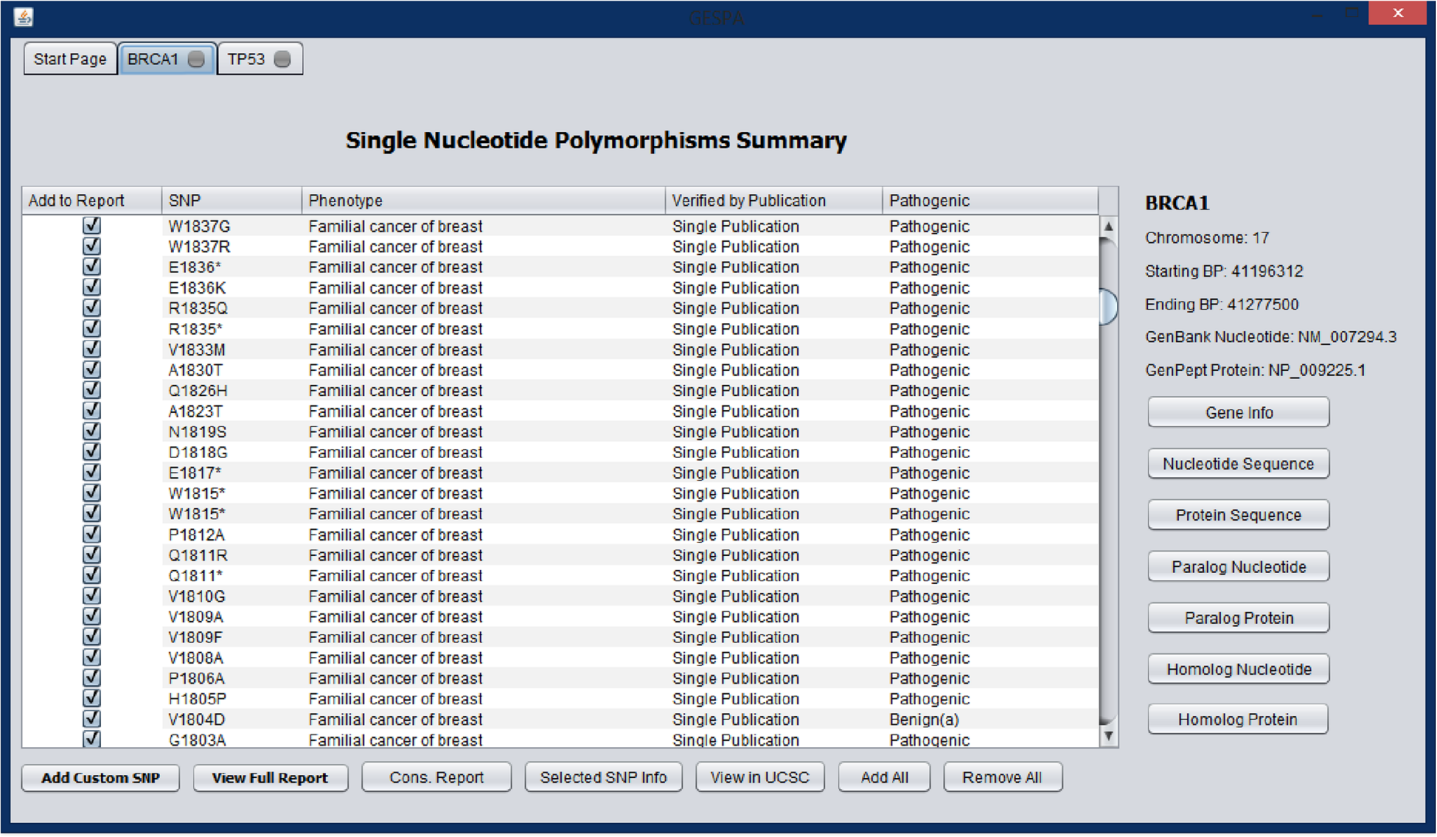
GESPA Main Interface. The gene summary interface allows access to important nsSNP and gene annotations. General information on the gene of the selected nsSNP is provided in the top right corner. Access to other important information related to the gene including nucleotide and protein sequences and alignments is located on the right side. Alignments and sequences open in separate closable tabs while gene info opens the corresponding page for the gene on NCBI. Access to annotations related to SNPs is found in the lower portion of the interface. Predictions of SNP phenotype and pathogenicity are displayed on the main summary table

One of our primary goals is to construct a user friendly interface for quick and efficient nsSNP analysis (Fig. 2). The tabbed interface allows for easy access to all genes and reports generated by GESPA. Parallel processing of different genes and their associated nsSNPs is fully supported and each batch and gene is processed on a separate tab. A table displays the most important information related to each nsSNP including protein location, predicted phenotype, number of related publications, and pathogenicity. Pathogenicity is indicated in three distinct ways. Benign (h) indicates that the nsSNP was found to be benign due to no nearby pathogenic nsSNPs identified in the literature, i.e., the nsSNP is likely not in a functional hotspot. Benign (a) indicates that the nsSNP was found to be benign by a low PSIC Score and/or WPC score, i.e., low conservation in alignment). Pathogenic indicates that the nsSNP is predicted to be pathogenic based on high WPC Score or a stop-gained mutation.

We offer a comprehensive conservation report to facilitate analysis of the genomic context surrounding a nsSNP of interest. Users can choose to add as many nsSNPs as they desire to a conservation report. This conservation report gives specific detail regarding the percentage of the sequence conserved in paralogous and orthologous alignments of both nucleotides and amino acids. Nucleotide and protein sequences for the gene in question as well as alignments of these sequences with paralogous and orthologous genes can be accessed in new tabs. The summary tables and the conservation tables provided by GESPA also include information on factors that could be used to predict the pathogenicity. These include the WPC Score, the PSIC score, the BLOSUM 62 substitution score [38], and literature near the nsSNP of interest.

For the collection of relevant data we provide external links related to each individual nsSNP and the entire gene. Gene information is accessed through its Entrez gene entry which contains information such as publications and links to other relevant databases. Viewing selected nsSNP information takes users to the associated ClinVar page which includes direct links to prior publications associated with the nsSNP and other related information in databases such as dbSNP and OMIM. Viewing the nsSNP results in a BLAT search opens the UCSC genome browser [39] to the location identified by the BLAT search. The UCSC interface allows users to see the nsSNP in the context of its location in the human genome and view the multitude of annotations collected by UCSC and others.

## Results and discussion

### Dataset summary

The humsavar, ClinVar and humvar datasets were used for the purpose of testing and training the pathogenicity and phenotype prediction algorithms of GESPA. The humsavar set consists of 62277 nsSNPs classified as either disease-associated (24399) or simple polymorphisms (37878). Humsavar incorporates data from UniProt [40] and contains likely phenotypes for all disease-associated nsSNPs that are part of the dataset. Analysis of the humsavar dataset showed that 584 benign nsSNPs were confirmed pathogenic in literature elsewhere.

The ClinVar dataset contains 22733 nsSNPs with predictions for pathogenicity and phenotype. Out of 22733 nsSNPs, 800 matched with those on humsavar. Even with this minimal overlap GESPA performed well when tested on humsavar.

Finally, in order to further confirm the accuracy of GESPA, the independent humvar [41] dataset was used as a training set for pathogenicity. The optimal cutoff values for GESPA’s pathogenicity prediction algorithm derived from training on humvar were tested on the humsavar dataset. The humvar dataset contains a total of 21185 nsSNPs, 8241 identified as polymorphisms and 12944 identified as pathogenic. Additionally, the humvar dataset shares 5333 nsSNPs with humsavar which were removed while assessing accuracy of GESPA. The humvar dataset also shares 1672 nsSNPs with ClinVar.

### Determination of pathogenicity classifiers

The WPC score, PSIC score, and BLOSUM 62 substitution score were considered as sequence level pathogenicity classifiers. In order to find which combination of the above classifiers would yield the greatest sensitivity and specificity, a nominal receiver operating characteristic (ROC) curve was created for each pathogenicity classifier using the humsavar dataset. Particularly, the optimal binary prediction of each of the individual classifiers was assessed against humsavar pathogenicity predictions. We found that both WPC and PSIC scores but not Blosum 62 score were good independent predictors of pathogenicity (WPC and PSIC: p < .0001 by the chi squared test, WPC AUC = 0.75912, PSIC AUC = 0.70863, Blosum 62: p = 0.3122, AUC = 0.64912). WPC Score indicates a greater conservation of the human allele in both orthologs and paralogs and therefore protein changes at the indicated location are more likely to be pathogenic. Unlike WPC, the PSIC algorithm [9] calculates the probability that an amino acid substitution will be tolerated at a specific position in an alignment [42] and was previously shown to be a good predictor of nsSNP pathogenicity [1, 8, 43]. In these studies the PSIC score was either integrated into a predictive algorithm for nsSNP pathogenicity [1, 8] or independently assessed as a single attribute [43]. The PSIC score differs from the WPC Score in the scope of data used; rather than predicting the risk of a detrimental amino acid change by assessing conservation in a single position in the alignment, the PSIC score attempts to take into context conservation throughout the alignment to make a prediction.

Next, the determination of whether a nsSNP could be in a functional hotspot using ClinVar literature was assessed as an independent boolean classifier and found to be highly effective in predicting pathogenicity of nsSNPs (p < 0.0001 by the chi-squared test).

### Pathogenicity prediction pipeline

GESPA was used to predict pathogenicity of nsSNPs using disease association information from ClinVar and thresholds for the WPC and PSIC scores. GESPA’s pathogenicity prediction algorithm is based on a step-wise decision making pipeline. First, disease association information from ClinVar is used so that nsSNPs not in functional hotspots are classified as benign (99.4 % of nsSNPs from humsavar were correctly predicted to be benign). To predict the pathogenicity of the nsSNPs which are in potential functional hotspots we used additional metrics. Particularly, the previously published PSIC score and our newly developed WPC score were employed to evaluate the sequence similarity around the nsSNP of interest. The PSIC score was found to be better than the WPC score in classifying benign nsSNPs (at maximum balanced accuracy when used alone on nsSNPs in potential functional hotspots, 91.6 % and 79.3 % of SNPs predicted benign by PSIC and WPC respectively were actually benign). The WPC score was still found to be a more accurate predictor of pathogenic nsSNPs (at maximum balanced accuracy when used alone on nsSNPs in potential hotspots, 61.4 % and 52.4 % of SNPs predicted pathogenic by WPC and PSIC respectively were actually pathogenic). Thus, literature from ClinVar is first used to classify nsSNPs not in functional hotspots as benign, then the PSIC score (if used, not included in default setting) is used to classify a nsSNP as benign, and finally the WPC score classifies remaining nsSNPs as either benign or pathogenic.

### Pathogenicity prediction accuracy

**Fig. 3:**
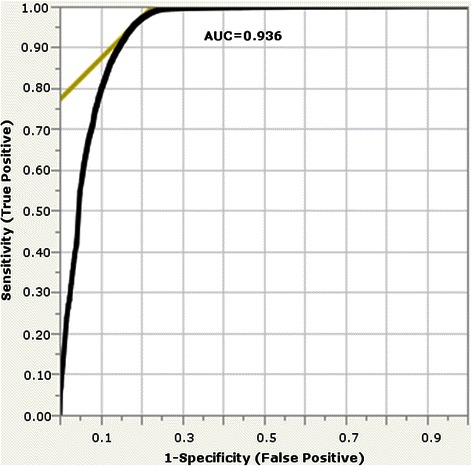
Multinomial ROC Curve for 5-fold cross validation on humsavar dataset. The multinomial ROC curve (black curve) is an average of 5 ROC curves which each represent the pathogenicity prediction accuracy of GESPA after training on four folds of humsavar and testing on the remaining fold. The WPC Score, PSIC Score, and nsSNPs for literature were used as predictors for pathogenicity. Using the point of maximum balanced accuracy (intersection of yellow line and black curve) for each curve, the optimal cutoff points of the WPC and PSIC Score for each fold could be determined and the AUC was found to be 0.936

The accuracy of GESPA’s pathogenicity prediction was calculated using a 5-fold cross validation of the humsavar dataset (Fig. 3). Additionally, the accuracy was tested on an independent humsavar dataset by using the humvar dataset [41] as a training set (Table 1). In both tests of GESPA’s pathogenicity prediction accuracy was similar (87.95 for humsavar cross validation and 87.78 for humsavar test set).

The cross validation was carried out by creating 5 different (approximately) equal sets of nsSNPs from humsavar by randomizing gene name in order to ensure that each set had no protein sequences in common (all the nsSNPs for a given gene were within the same test fold) and therefore each test fold would be evaluated for the values of cutpoints that had been optimized for a training set (composed of the other four folds) that contained separate protein sequences and nsSNPs (Additional file 1: Table S1). Furthermore, even nsSNPs with identical amino acid changes on differing genes would not necessarily be similar due to GESPA’s approach of using conservation and gene-specific literature. The accuracy of the cross validation and the values for the cutpoints were obtained by averaging the respective values obtained in 5 iterations. Moreover, new cutpoints from training on humvar were used to estimate the accuracy of the pathogenicity prediction pipeline on the humsavar dataset. Note that, the 5333 redundant nsSNPs between humsavar and humvar were removed in order to prevent potential bias.

The overall WPC and PSIC cutpoints for the final pathogenicity algorithm were obtained by averaging the cutpoints obtained by both the procedures. The PSIC algorithm yielded a cut point similar to those found by cross validation procedures performed by Ramensky *et al*., Adzhubei *et al*. and Dobson *et al.* [1, 8, 43]. We found that in the combined algorithm, the highest accuracy of pathogenicity predictions is possible when nsSNPs with a PSIC score of less than 1.03 or a WPC less than 40 are predicted benign and nsSNPs with a WPC score of greater than or equal to 40 are predicted pathogenic.

In GESPA’s default setting the use of PSIC Score is turned off for computational simplicity. The cutpoints for this case were determined separately using the similar procedure described above with the exclusion of the PSIC score. The optimal cutpoint for the WPC score alone was found to be 49. In other words, the nsSNPs not classified benign with functional hotspot/literature analysis that have a WPC ≥ 49 were predicted pathogenic in default settings of GESPA. Note that the accuracy of the algorithm when using WPC score in the absence of PSIC score was lower (sensitivity = 94.83, specificity = 80.42, balanced accuracy = 87.62) than the results on Table 1. The software automatically applies the cutpoints for WPC when the PSIC score option is not selected.

### Phenotype prediction

The phenotype of a given nsSNP is predicted by GESPA by using the literature containing nsSNPs in a search and ranking type process. Particularly, GESPA determines the phenotype of the nsSNP by evaluating association of nsSNPs identified within a user-specified range of amino acids towards the 3′ and 5′ ends (100 is the default). The phenotype frequently associated with the nsSNPs in the given range is predicted to be the phenotype of the nsSNP. Combining the literature information on multiple nsSNPs in a given range reduces the bias induced by frequent and possibly contradictory annotations of one nsSNP. If the relevant information is not available the program is unable predict a phenotype.

To test the accuracy of the phenotype predictions, the phenotype predictions of 1080 nsSNPs were manually compared to the phenotype annotation on the humsavar database which served as a ground truth. The nsSNPs in question were obtained by randomizing the order of genes in the humsavar dataset and choosing the first 1080 nsSNPs in these genes with a phenotype (Additional file 2: Table S2). The correct phenotype was predicted with an accuracy of 96 %. In 170 cases GESPA was unable to predict phenotypes due to a lack of data (Table 2). Within this group of 170, 79 phenotypes were predicted to have high functional significance, but a specific condition was not predicted because nearby nsSNPs were only annotated as functionally significant. Note that phenotype prediction accuracy cannot be expressed in terms of sensitivity or specificity.

**Table 2:** GESPA phenotype prediction for randomized sample of 1080 nsSNPs

Data not available (15.7 %)		Data available (84.3 %)	
Correct Phenotype, less accurate prediction	79 (46 %)	Correct Prediction	870 (96 %)
No SNPs on Gene in given range	73 (43 %)	Incorrect Prediction	40 (4 %)
No previous SNPs on Clinvar	18 (11 %)		
Overall Accuracy: 80.56 %			

### Comparison with existing tools

GESPA combines four distinct algorithms into its stepwise pathogenicity classification in the following order: (1) Analysis of whether an nsSNP results in stop-gained mutation, (2) Reports of pathogenicity of other nsSNPs in the same gene as obtained from ClinVar, (3) PSIC Score, and (4) WPC Score. Currently available programs for predicting nsSNP pathogenicity are limited in clinical application by a lower than acceptable sensitivity. GESPA achieved a sensitivity of 96.3 % and specificity greater than 79.3 % which exceeds current standards based on existing tools, making it feasible to use in direct clinical applications. Particularly, GESPA’s performance was assessed by using the procedure and data-sets published in Choi *et al.* [37] to compare existing programs (Table 1). Hence, we could directly compare sensitivity, specificity, balanced accuracy, and the AUC of the GESPA ROC curve with the same parameters for existing programs published by Choi *et al.* [37], to find that GESPA exceeds all of these values.

Furthermore, while GESPA has a balanced accuracy greater than other programs by 9.20-12.39 %, the dramatic increase in sensitivity over these programs is even more pronounced at 7.73-18.02 %. GESPA has a very low percentage of false negatives (3.69 %) and a high balanced accuracy (87.95 %, Table 1), leading us to believe that it can be implemented in many clinical applications.

The determination of whether a nsSNP is not in a functional hotspot using medical literature found in ClinVar is one of the most important reasons the pathogenicity prediction accuracy of GESPA exceeds existing programs. To our knowledge, no other existing software package has incorporated medical literature into an nsSNP pathogenicity prediction algorithm. Particularly, the nsSNP phenotype data from ClinVar is used in order to correctly classify benign mutations not located in functional hotspots before those that may be located in the functional hotspots. Moreover, to deal with the limited availability of the data in ClinVar and to mitigate potential bias of sampling arising only from the use of medical literature information, GESPA extends the phenotype data used from ClinVar by implementing the WPC score, PSIC score and analysis of whether nsSNPs results in nonsense mutations. The user may choose whether information from ClinVar or the PSIC algorithm is used in the pathogenicity prediction algorithm, but both provide an increase in the overall pathogenicity prediction accuracy.

### Humsavar discrepancies

Ideally the training sets should act as a perfect gold standard, but our investigation of humsavar has shown otherwise. Although the dataset was found to be one of the most accurate datasets reflecting nsSNPs available by cross validation and random sampling [44], it is based on literature reports which would make classification of benign mutations difficult due to a possible lack of literature. GESPA’s greatest potential for improvement is in its pathogenicity classification specificity. In light of this, it is possible that many more of the false positives identified by GESPA could in fact be true positives (see testing and training datasets). However, the idea of false negatives actually being true negatives is not as likely. The false negatives would have had to be misidentified by literature, an unlikely possibility.

### Future directions

Currently GESPA fully supports the latest version of the human genome and will continue to update to the latest version of the genome available. GESPA does not support animal genomes although information on animal genes orthologous to human genes is available in the program. Allowing analysis of animal nsSNPs has great potential and will be the focus of future work. In addition, while GESPA is currently available as a standalone software, future editions will also allow limited functionality within a webpage.

In future versions of GESPA we will integrate several annotations that could not be added due to a lack of available data. Information related to potential race and ethnicity associated disease risk based on specific SNPs is currently available on ClinVar for a very limited number of SNPs. However, as more information becomes available, GESPA may be able to integrate this information for improving its prediction. The same idea may also be applied to disease penetrance and whether SNPs are expressed in homozygous or heterozygous contexts. Application of GESPA to specific case studies, often of clinical origin, will also be the focus of future work.

## Conclusions

GESPA is a unique software package for classifying nsSNPs. GESPA exceeds the accuracy of other programs in its class and has high sensitivity. The software is the first to predict the phenotype of SNPs in addition to pathogenicity. Furthermore, GESPA offers several additional features such as a variety of annotations, parallel processing, fast SQL cloud data retrieval, and a user friendly interface. We believe that GESPA provides researchers and healthcare providers with great utility for analyzing the disease association of nsSNPs comprehensively.

## Availability and requirements

**Project Name:** GESPA

**Project home page:** www.sourceforge.net/projects/gespa

**Operating system:** Windows: XP SP3 or later, Windows Vista SP2, Windows 7, Windows 8, Mac OS X 10.7.3 (Lion) or later

**Programming language:** Java

**Other requirements:** Java 1.7.0 or higher

**License:** GPL 3.0 License, Apache License 2.0, Microsoft Reciprocal License

## Additional files

Additional file 1: Table S1. Cross Validation Folds: Spreadsheets containing all 5 folds, for GESPA’s cross validation process. Also includes the numerical values of GESPA’s pathogenicity and phenotype classifiers for every nsSNP within each test fold. For the cross validation the five different combinations of four folds were used to train GESPA’s pathogenicity classifiers and the remaining fold in each case was used to test the optimal parameters. 
Additional file 2: Table S2. Phenotype Prediction Sample: Known (from humsavar) and predicted (by GESPA) phenotypes of 1080 nsSNPs.

## Abbreviations

nsSNPs: Non-synonymous single nucleotide polymorphisms
GESPA: GEnomic Single nucleotide Polymorphism Analyzer
PSIC: Position Specific Independent Counts
NGS: Next generation sequencing
WPC: Weighted Protein Conservation
